# Special role of *JUN* in papillary thyroid carcinoma based on bioinformatics analysis

**DOI:** 10.1186/s12957-017-1190-8

**Published:** 2017-07-03

**Authors:** Wenzheng Chen, Qingfeng Liu, Yunxia Lv, Debin Xu, Wanzhi Chen, Jichun Yu

**Affiliations:** 1 Department of Thyroid and Neck Surgery, The Second Affiliated Hospital of Nanchang University, Nanchang, Jiangxi Province 330006 China; 2 Department of General Surgery, The People’s Hospital of Liaoning Province, Shenyang, 110016 China

**Keywords:** PTC, DEGs, Enrichment analysis, Interaction network, *JUN*

## Abstract

**Background:**

Papillary thyroid carcinoma (PTC) is the most common malignancy in thyroid tissue, and the number of patients with PTC has been increasing in recent years. Discovering the mechanism of PTC genesis and progression and finding new potential diagnostic biomarkers/therapeutic target genes of PTC are of great significance.

**Methods:**

In this work, the datasets GSE3467 and GSE3678 were downloaded from the Gene Expression Omnibus (GEO) database. Differentially expressed genes (DEGs) were identified with the limma package in R. GO function and KEGG pathway enrichment were conducted with DAVID tool. The interaction network of the DEGs and other genes was performed with Cytoscape plugin BisoGenet, while clustering analysis was performed with Cytoscape plugin ClusterOne.

**Results:**

A total of 1800 overlapped DEGs were detected in two datasets. Enrichment analysis of the DEGs found that the top three enriched GO terms in three ontologies and four significantly enriched KEGG pathways were mainly concerned with intercellular junction and extracellular matrix components. Interaction network analysis found that transcription factor hepatocyte nuclear factor 4, alpha (HNF4A) and DEG *JUN* had higher connection degrees. Clustering analysis indicated that two function modules, in which *JUN* was playing a central role, were highly relevant to PTC genesis and progression.

**Conclusions:**

*JUN* may be used as a specific diagnostic biomarker/therapeutic molecular target of PTC. However, further experiments are still needed to confirm our results.

## Background

The thyroid cancer incidence has been increasing worldwide in recent years, and more cases of thyroid cancer are diagnosed every year [1]. Papillary thyroid carcinoma (PTC) is the most common malignancy in the thyroid and accounts for almost 80% of all thyroid cancers [2]. It is characterized by distinctive nuclear alterations including pseudoinclusions, grooves, and chromatin clearing [3]. Most patients with PTC have an excellent prognosis, but a small number of patients remain suffering with aggressive PTC which can develop invasive tumors and/or distant metastases [4]. Undoubtedly, PTC places an enormous economic burden on society and personality and greatly lowers the quality of one’s life. It is of great significance to study the mechanism of PTC genesis and explore new avenues to prevent PTC formation.

Previous study indicated that a number of different genetic changes were related to PTC, particularly the chimeric oncogenes formed by a fusion of a membrane receptor protein tyrosine kinase domain with another gene’s 5-prime terminal region. Oncogenic gene rearrangements involving the RET and NTRK1 have been found in PTC tissues [5, 6]. BRAF and RAS mutations are also observed in PTC cases, and the constitutive activation of effectors along the RET/PTC-RAS-BRAF signaling pathway contributed to the transformation of the thyroid cell to PTC [7–9]. For the diagnosis and prognosis of PTC, several methods and markers are used. Immunohistochemical markers have been evaluated and tested in PTC tissues, such as CK19, HBME-1, RET, galectin-3, and CITED1. However, they are helpful only in some cases, for all of them have their limits and may bring some error diagnostics [10–13]. Molecular studies also conferred some useful information for the diagnosis and therapy of PTC. Liu et al. have reported that CXCR7 gene involves in regulating proliferation and metastasis of PTC cell and provides a potential target for therapeutic interventions in PTC [14]. Minna et al. found that miR-199a-3p could act as a tumor suppressor in PTC [15]. Despite those researches on PCT, there are still so many mechanisms underlining PTC genesis and progression that needed further investigation, especially in the gene expression profile level.

In this study, we aimed to identify the differentially expressed genes (DEGs) in PTC tissues compared with normal thyroid tissue adjacent to PTC tumors. A series of bioinformatics analyses including DEGs identification, function enrichment, and interaction network construction were conducted to gain more insights into the molecular mechanisms of PTC genesis and progression. Our aim is to explore the pathogenesis of PTC and find potential diagnostic biomarkers/therapeutically targets of PTC by bioinformatic methods.

## Methods

### Affymetrix microarray data

Gene expression profiles of GSE3467 and GSE3678 were downloaded from the Gene Expression Omnibus (GEO) database (http://www.ncbi.nlm.nih.gov/geo/), which is sequenced on the GPL570 (Affymetrix Human Genome U133 Plus 2.0 Array) platform. A total of 32 chips were used for the analysis, including 18 samples in GSE3467 (9 PTC samples and 9 matched normal tissue samples [16]) and 14 samples in GSE3678 (7 PTC samples and 7 paired normal thyroid tissue samples). Total RNA was extracted from paired tumor and normal thyroid tissues from the PTC patients. The downloaded raw data in CEL files were converted into expression measures and performed background correction and quartile data normalization using the robust multichip average (RMA) algorithm [17] in Affy package manufactured by Affymetrix [18].

### DEGs analysis

Both aforementioned datasets were divided into the PTC group and the normal group. The limma method [19] was used to identify DEGs in both datasets. The threshold of DEGs was set as |log_2_FC| > 0.5 with false discovery rate (FDR) <0.01. The BH method was used to adjust the raw *p* value into FDR to circumvent the multi-test problem which might induce too many false positive results [20]. Subsequently, two sets of DEGs were obtained after the above process. Venn diagram package [21] was used to perform Venn diagram to get the overlapped DEGs in both datasets. To further study the overlapped DEGs, heat maps of the overlapped DEGs were depicted in both datasets using R package “pheatmap” function [22]. We could inspect the different expression patterns of these genes between the PTC group and the normal group through the heat maps. Besides, the correlation analysis between the logFC values of the DEGs in GSE3467 and GSE3678 was also processed to verify whether the gene expression trends in both datasets were the same or not [23]. The Pearson’s correlation coefficient was used to assess the associations. All *p* values <0.05 were considered to be statistically significant.

### Enrichment analysis

Firstly, the probes were converted to the official gene symbol according to Da et al. [24] using DAVID. Then, both Gene Ontology (GO) enrichment analysis and Kyoto Encyclopedia of Genes and Genomes (KEGG) pathway enrichment analysis were processed to complete the functional enrichment analysis, and DAVID was utilized to select online biological classification. GO provides three structured networks of defined terms (biological process, molecular function, and cellular compartment) to describe gene product attributes [25]. We performed GO enrichment analysis in these three ontologies to functionally classify the DEGs. KEGG pathway is a collection of manually drawn metabolic pathway map which represents our knowledge on the molecular interaction and reaction networks [26]. The count number ≥5 and the *p* value <0.01 were chosen as the cutoff for defined GO terms and KEGG pathways.

### Interaction network construction

Cytoscape [27] is a free software project for integrating biomolecule interaction networks with high-throughput expression data and other molecular states into a unified conceptual framework. BisoGenet [28] is a new plugin of Cytoscape for gene network construction, visualization, and analysis. A trait of Bisogenet is the availability to include coding relations to distinguish between genes and their products. In the present study, BisoGenet was used to get the interaction networks of the DEGs based on Biomolecular Interaction Network Database (BIND) [29], a database designed to store full descriptions of interactions, molecular complexes, and pathways, to search the all known interactions. The interaction patterns with a degree ≥0.8 were selected. Besides, the clustering analyses of the genes were also performed with the Cytoscape plugin ClusterOne [30] under default parameters to obtain the important function modules, and the *p* values of hypergeometric distribution were defined <0.05.

## Results

### DEGs analysis

Totally, 4237 DEGs were identified in GSE3467, and 2990 DEGs were identified in GSE3678. What's more, there were 1800 overlapped DEGs observed in both datasets. Among them, 1083 genes were significantly downregulated while 717 genes were significantly upregulated.

**Fig. 1:**
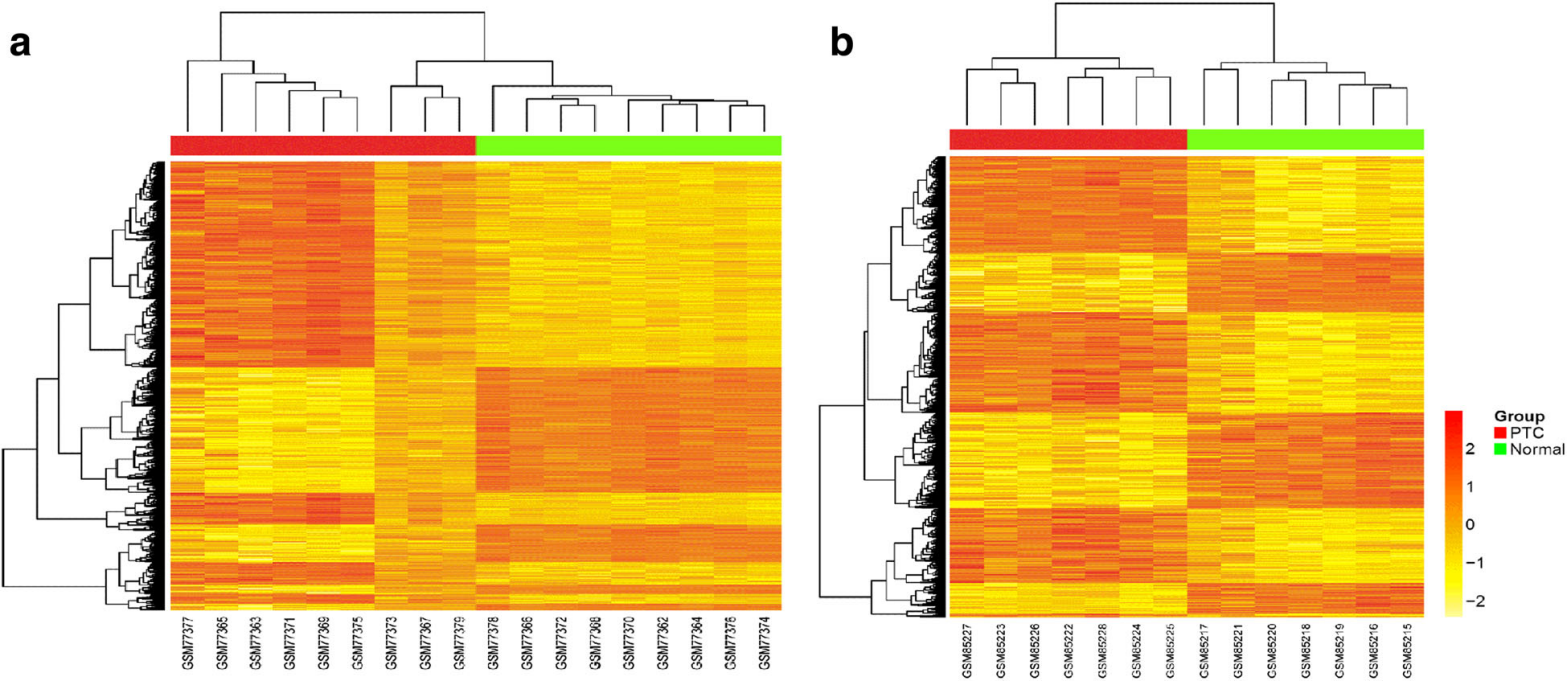
Bidirectional hierarchical clustering analysis of the 1800 overlapped DEGs between PTC and control groups in dataset GSE3467 (**a**) and GSE3678 (**b**)

**Fig. 2:**
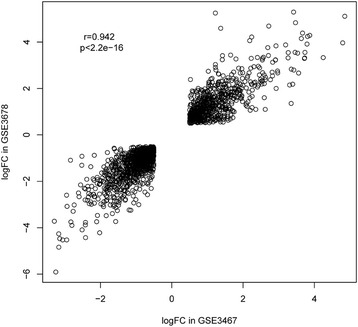
Correlation scatter plot of the 1800 DEGs’ logFC values in dataset GSE3467 and that in dataset GSE3678

The hierarchical clustering analysis of the 1800 overlapped genes in the two datasets is shown in Fig. 1. As the heat maps have shown, in both datasets, the expression patterns of these genes were significantly different between the PTC group and the control group, and there was an obvious boundary line between them. Besides, correlation analysis between the logFC values of the DEGs in GSE3467 and GSE3678 also showed that they were highly positively correlated as the correlation coefficient reached 0.94 and the *p* value <2.2e − 16 (Fig. 2). This result indicated that the expression patterns of the DEGs in the two datasets were highly consistent, and the 1800 overlapped DEGs may be truly differentially expressed in the PTC group compared with those in the control group.

### Function enrichment analysis

A total of 179 significantly enriched GO terms in the aforementioned three ontologies and four significantly enriched KEGG pathways were obtained. The top three enriched GO terms in the three ontologies and the four enriched KEGG pathways are listed in Table 1. Taken together, the intercellular junction and extracellular matrix components may be related with the PCT genesis.

**Table 1:** The top five enriched GO terms in three categories and all enriched KEGG pathways in PTC

Category	Term	*P* value
GOTERM_BP_FAT	GO:0007155~cell adhesion	8.71E-08
GOTERM_BP_FAT	GO:0022610~biological adhesion	9.42E-08
GOTERM_BP_FAT	GO:0009611~response to wounding	1.53E-06
GOTERM_BP_FAT	GO:0007242~intracellular signaling cascade	1.92E-06
GOTERM_BP_FAT	GO:0008637~apoptotic mitochondrial changes	2.35E-06
GOTERM_CC_FAT	GO:0031012~extracellular matrix	4.21E-07
GOTERM_CC_FAT	GO:0044459~plasma membrane part	7.73E-06
GOTERM_CC_FAT	GO:0005578~proteinaceous extracellular matrix	7.77E-06
GOTERM_CC_FAT	GO:0044421~extracellular region part	1.17E-05
GOTERM_CC_FAT	GO:0000267~cell fraction	3.87E-05
GOTERM_MF_FAT	GO:0030247~polysaccharide binding	1.49E-05
GOTERM_MF_FAT	GO:0001871~pattern binding	1.49E-05
GOTERM_MF_FAT	GO:0042802~identical protein binding	5.24E-05
GOTERM_MF_FAT	GO:0008092~cytoskeletal protein binding	5.74E-05
GOTERM_MF_FAT	GO:0005539~glycosaminoglycan binding	7.06E-05
KEGG_PATHWAY	hsa04512:ECM-receptor interaction	1.05E-04
KEGG_PATHWAY	hsa05200:Pathways in cancer	1.20E-04
KEGG_PATHWAY	hsa05210:Colorectal cancer	3.07E-03
KEGG_PATHWAY	hsa04510:Focal adhesion	3.28E-03

### Interaction network analysis

**Fig. 3:**
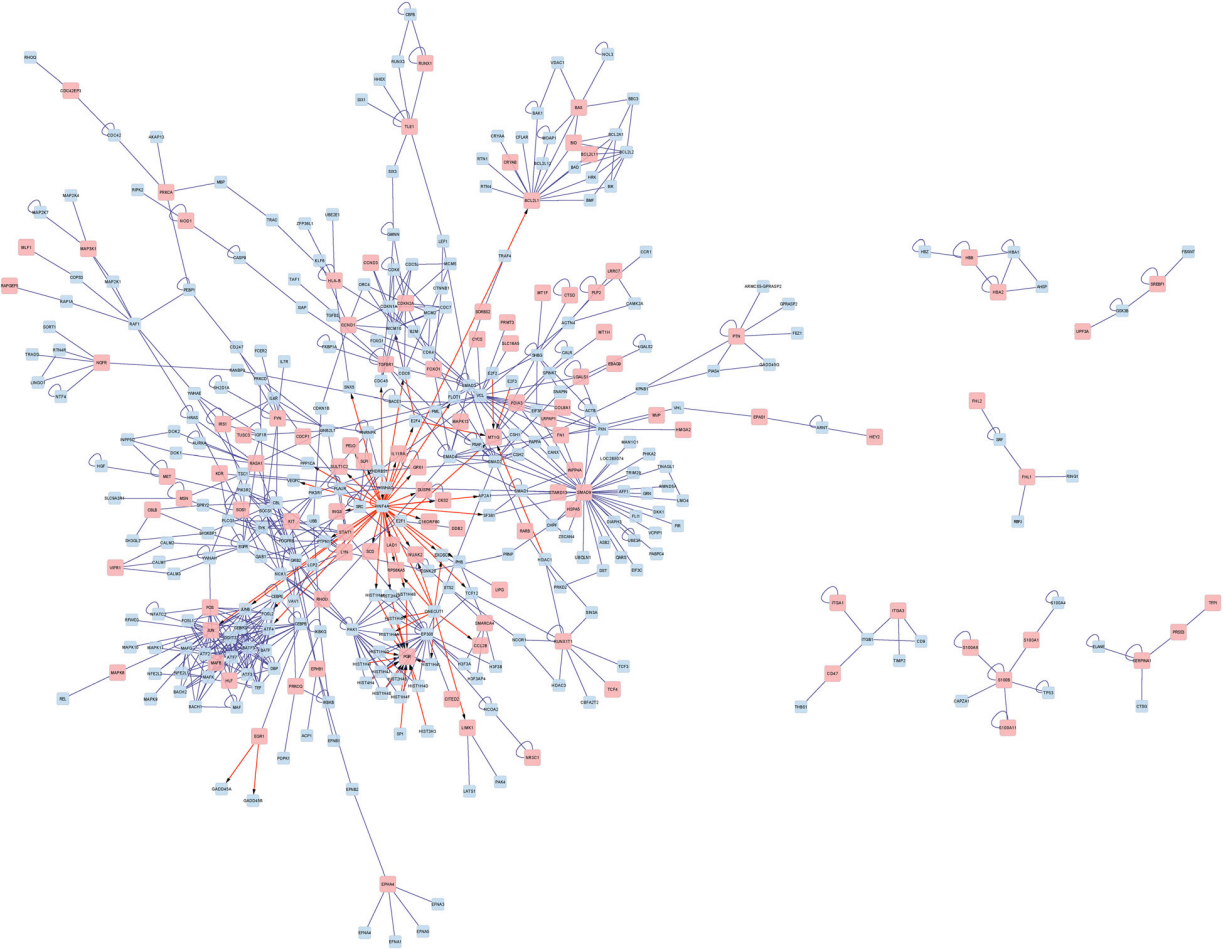
DEGs interaction network construction in PTC. The *red squares* stand for DEGs and the *blue squares* stand for target proteins. The *blue lines* stand for the interaction between two proteins and the *blue lines* with *arrows* stand for the interaction between DNA and protein

**Fig. 4:**
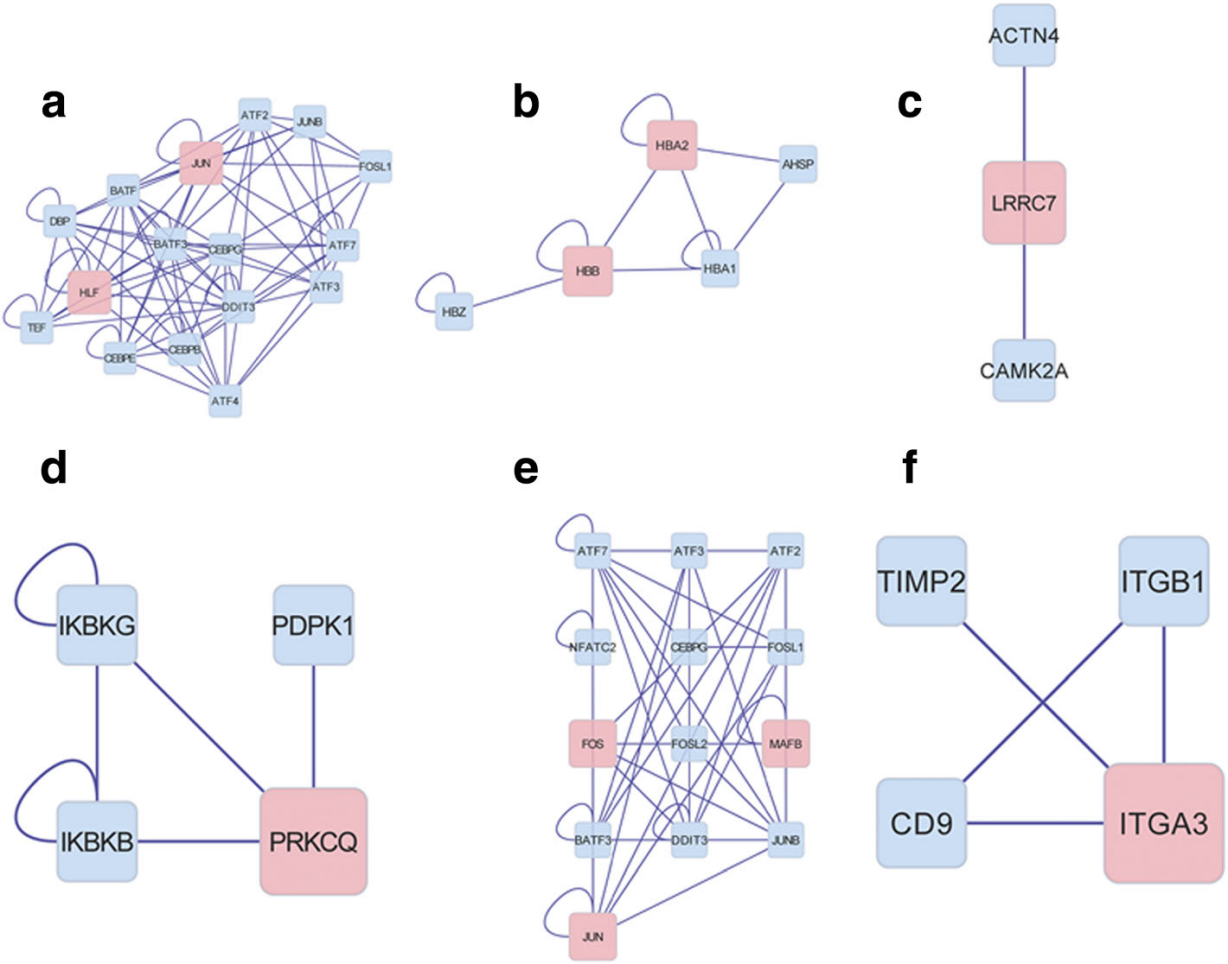
Significant clusters in PTC. The interaction networks in cluster 1 (**a**), cluster 2 (**b**), cluster 3 (**c**), cluster 4 (**d**), cluster 5 (**e**), and cluster 6 (**f**)

The interaction network of the DEGs was created to deeply understand how these DEGs are related and how the different pathways crosslink to each other (Fig. 3). As the result had shown, transcription factor (TF) hepatocyte nuclear factor 4, alpha (HNF4A), regulated the expression of many DEGs, while the DEG *PGR* could be regulated by many transcription factors. The DEG *JUN* was connected with many other genes, which implied that its encoding product could interact with many target proteins, and be involved in many pathways. Six function clusters of the DEGs were obtained using the plugin ClusterOne including cluster 1 with the DEGs *JUN* and *HLF* (Fig. 4a), cluster 2 with the DEGs *HBA2* and *HBB* (Fig. 4b), cluster 3 with the DEG *LRRC7* (Fig. 4c), cluster 4 with the DEG *PRKCQ* (Fig. 4d), cluster 5 with the DEGs *JUN*, *FOS*, and *MAFB* (Fig. 4e), and cluster 6 with the DEG *ITGA3* (Fig. 4f). These six significant clusters mainly function in protein dimerization, hemoglobin complex, cytoplasmic vesicle, regulation of molecular function, sequence-specific DNA binding, and integrin complex. The detailed genes involved in these clusters and the top significant GO term of these genes were listed in Table 2. What was noticeable was that the gene *JUN* appeared in two clusters (cluster 1 and cluster 5), suggesting its important role in PTC genesis.

**Table 2:** The detailed information of the significant enriched function modules

	Nodes	*P* value	Genes	Top significant GO term
Cluster 1	16	0.0000143	ATF2, ATF3, ATF4, BATF, CEBPE, HLF, DBP, TEF, JUN**,** CEBPB, BATF3, DDIT3, JUNB, CEBPG, FOSL1, ATF7	GO:0046983~protein dimerization activity
Cluster 2	5	0.005	HBZ, HBB, HBA1, HBA2, AHSP	GO:0005833~hemoglobin complex
Cluster 3	3	0.026	CAMK2A, ACTN4, LRRC7	GO:0044433~cytoplasmic vesicle part
Cluster 4	4	0.027	IKBKB, IKBKG, PRKCQ, PDPK1,	GO:0044093~positive regulation of molecular function
Cluster 5	13	0.032	ATF2, ATF3, NFATC2, MAFB, JUN, FOSL2, BATF3, DDIT3, JUNB, CEBPG, FOS, FOSL1, ATF7	GO:0043565~sequence-specific DNA binding
Cluster 6	4	0.05	CD9, ITGA3, ITGB1, TIMP2	GO:0008305~integrin complex

## Discussion

The incidence of PTC has increased worldwide over the past 15 to 20 years, especially in developed countries [31, 32]. To manage the increasing PTC patients effectively, a better understanding of the molecular mechanism involved in PTC is necessary. We applied bioinformatics techniques to investigate the DEGs in PTC and deeper explore the molecular mechanism underlying PTC genesis in this study. A total of 1800 overlapped DEGs were detected in two datasets. Enrichment analysis found the top five enriched GO terms in three ontologies and four significant enriched KEGG pathways were mainly concerned with regulation of pigmentation, cellular homeostasis, extracellular matrix, and intercellular junction. By constructing interactive network of the DEGs, we found that transcription factor HNF4A and DEG *JUN* had higher connection degrees in the network. By employing plugin ClusterOne, we got six subnetworks and DEG *JUN* appeared in two subnetworks.

In the constructed interaction network, we observed that the transcription factor HNF4A was connected with many other DEGs. However, its own expression has not changed in PTC. HNF4A regulates expression of genes involved in glucose metabolism and homeostasis [33]. It may play important roles in the occurrence and progression of PTC by interfering other genes’ normal expression and further disturbing cellular homeostasis. Nevertheless, further studies are still needed to illustrate its specific role in PTC genesis. We also discovered that the DEG gene *JUN* could interact with many other target proteins, implying its important role in PTC genesis. *JUN* is a proto-oncogene, and its encoding product is the first discovered oncogenic transcription factor [34]. Previous study demonstrated that it could promote tumor formation and maintain tumor cell survival between the initiation and progression stages [35]. The activation of *JUN* was also involved in the progress of breast cancer, gastric cancer, and colorectal carcinomas [36–38]. But the role of *JUN* in PTC has not been studied, and here, we observed its significant different expression in PTC tissues and noticed its interaction with so many other proteins in the created interaction network. We deduced that the *JUN* may also play an important role in PTC genesis and progression and can be a possible potential diagnostic biomarker/therapeutical target gene of PTC.

Our deduction get further conferred by the cluster analysis, as the *JUN* appeared again in cluster 1 and cluster 5 (Fig. 4a, e). GO enrichment analysis manifested that cluster 1 was mainly concerned with “protein dimerization activity.” There were only two DEGs in cluster 1, namely *JUN* and *HLF*. The rest were all unchanged target proteins, and most of them were transcription factors, such as ATF2, ATF3, ATF4, ATF7, DDIT3, and FOSL1. *HLF* is a proto-oncogene whose expression product is a subset of the bZIP transcription factors and can cause abnormal transcriptional regulations of target genes which is related to leukemia development [39]. ATF2 binds with JUN to form a heterodimer and participates in reducing the amount of tumor necrosis factor (TNF) transcription through competitive binding [40]. The co-expressing JUN and ATF3 in two neuronal-like cell lines significantly enhanced JUN-mediated neurite sprouting [41]. The interactions of JUN with the aforementioned other transcription factors in regulation certain gene expression have also been reported [41, 42]. We presumed that the activated JUN might interact with some transcription factors and further induce abnormal gene expressions which lead to carcinogenesis.

The top significant GO term of function cluster 5 was “sequence-specific DNA binding.” Many transcription factors, such as ATF2, ATF3, ATF4, and ATF7, were also clustered to this module. What is noteworthy is the gene *FOS*, another proto-oncogene playing an important role in tumourigenesis and carcinogenesis [43], which changed its expression either in this module. The expression product of *FOS* can dimerize with JUN and form Activator Protein-1 (AP-1) complex. AP-1 binds to target genes at AP-1-specific sites at the promoter/enhancer regions and converts extracellular signals into changes of gene expression [44]. Some studies found that the JUN/FOS dimer, namely AP-1 complex, was involved in certain cancer genesis, and it can be the potential targeted therapeutic genes for certain cancer therapy. Magrisso et al. state that the expression of JUN and/or FOS are important events in colorectal tumorigenesis [45]. Wong et al. reported that the cyclooxygenase-2 inhibitor (SC-236) functioned the antitumor effects via inhibiting JNK-c-Jun/AP-1 activation, and the inhibition of JNK activation may have a therapeutic benefit against gastric cancer [37]. Zhang demonstrated that geldanamycin is a highly potent inhibitor of the AP-1 transcription factor and affects the activation of JNK in hypoxic HT29 human colon adenocarcinoma cells [36]. We supposed that the JUN/FOS dimer might also act as a promoter in PTC genesis. Design drugs targeting at this complex is potentially effective in PTC therapy. Even though, further immunohistochemical studies are still needed to confirm our results.

## Conclusions

In conclusion, we had analyzed the gene expression profiles of PTC using bioinformaticanalysis. Interaction network analysis indicated that the gene *JUN* was closely connected with PTC genesis. It might be used as specific therapeutic molecular target in order to benefit the cure of PTC patients. However, further experiments are still needed to confirm our results.

## Abbreviations

BIND: Biomolecular Interaction Network Database
DEGs: Differentially expressed genes
FDR: False discovery rate
GEO: Gene Expression Omnibus
GO: Gene Ontology
KEGG: Kyoto Encyclopedia of Genes and Genomes
PTC: Papillary thyroid carcinoma
TF: Transcription factor
TNF: Tumor necrosis factor
